# Novel Association of WNK4 Gene, Ala589Ser Polymorphism in Essential Hypertension, and Type 2 Diabetes Mellitus in Malaysia

**DOI:** 10.1155/2016/8219543

**Published:** 2016-05-29

**Authors:** Nooshin Ghodsian, Patimah Ismail, Salma Ahmadloo, Farzad Heidari, Polin Haghvirdizadeh, Sima Ataollahi Eshkoor, Ali Etemad

**Affiliations:** ^1^Genetic Research Group, Department of Biomedical Science, Faculty of Medicine and Health Sciences, Universiti Putra Malaysia, Serdang, Selangor, Malaysia; ^2^Iranian Research Center on Aging, University of Social Welfare and Rehabilitation Science, Tehran, Iran

## Abstract

With-no-lysine (K) Kinase-4 (WNK4) consisted of unique serine and threonine protein kinases, genetically associated with an autosomal dominant form of hypertension. Argumentative consequences have lately arisen on the association of specific single nucleotide polymorphisms of* WNK4* gene and essential hypertension (EHT). The aim of this study was to determine the association of Ala589Ser polymorphism of* WNK4* gene with essential hypertensive patients in Malaysia.* WNK4* gene polymorphism was specified utilizing mutagenically separated polymerase chain reaction (PCR) and restriction fragment length polymorphism (RFLP) method in 320 subjects including 163 cases and 157 controls. Close relation between* Ala589Ser *polymorphism and elevated systolic and diastolic blood pressure (SBP and DBP) was recognized. Sociodemographic factors including body mass index (BMI), age, the level of fasting blood sugar (FBS), low density lipoprotein (LDL), and triglyceride (TG) in the cases and healthy subjects exhibited strong differences (*p* < 0.05). The distribution of allele frequency and genotype of* WNK4 *gene* Ala589Ser* polymorphism showed significant differences (*p* < 0.05) between EHT subjects with or without type 2 diabetes mellitus (T2DM) and normotensive subjects, statistically. The* WNK4* gene variation influences significantly blood pressure increase.* Ala589Ser* probably has effects on the enzymic activity leading to enhanced predisposition to the disorder.

## 1. Introduction

Essential hypertension, as a significant health risk factor leading to renal and cardiovascular disorders, is a common disorder developed by unhealthy lifestyle habits and heritable elements. The genetic influence or heritability estimation on blood pressure (BP) variation displays remarkable range (30 to 50%) according to Timberlake et al. [1]. Genetic variations can considerably affect EHT genesis which significantly exhibits risk factor for progressive renal damage, stroke, ischemic heart disease, and peripheral vascular disease [2]. The specified genes are desired objectives for genetic, physiological, and functional research on EHT; however, these rare mutations do not restate BP variation in general population [3].

Previous prospective and case control studies have shown that hypertension progression is an independent predictor of T2DM [4]. Several possible factors are likely to be causes of the association between T2DM and EHT. Many reports have emphasized that patients with chronic diseases such as hypertension have diabetes [5]. The relationship between hypertension and diabetes also was obtained by other research groups [6].

The serine-threonine kinase with-no-lysine (K) 4 gene (MIM # 601844) is a hot locus for blood pressure that is on 17th chromosome [7]. In addition to the rare variants specification in WNK4 that is reliable for the pseudohypoaldosteronism type II, common single nucleotide polymorphisms (SNPs) in these genes have established relationship with BP variation and susceptibility to hypertension in general population in children and adults [8, 9]. The substitute of a nonpolar residue alanine by a polar residue serine can be produced by the Ala589Ser polymorphism as a missense mutation. According to Wilson and colleagues, mutations in WNK4 can cause some autosomal dominant Mendelian trait contributing to hypertension [10]. Recent studies also illustrate that WNK4 influences dominantly the distal convoluted tubule located in the kidneys as significant areas balancing reabsorption of water and salt leading to blood pressure determination [10–12]. According to Sun et al. [13], the WNK4 gene seems to influence considerably pathogenesis of essential hypertension. Ala589Ser polymorphism (as a missense mutation) might also make alterations to enzyme's functions, leading to increased susceptibility to the disorder. However, monogenic disorders are generally resulting from uncommon polymorphism in a gene coding sequence; usual disorders seem to be produced by genetic polymorphism in gene regulatory factors changing the locus expressional profile [14, 15].

Considering a strong relation between hypertension and diabetes in this study, our major target aimed at hypertension as well as T2DM to specify role of the gene underlying the disease to modify the prognostication of those at risk and also administer further antihypertensive treatments. The current research principal objective was to explore the relation of WNK4 gene Ala589Ser polymorphism between EHT and T2DM in Malaysian subjects. Several screening tests resulted in the recognition of human-specific variant Ala589Ser in WNK4 exon 8. This polymorphism (Ala589Ser) was targeted to be conducted on Malaysian population through evolutionary genetic analysis and to explore its relation with BP and FBS development as well.

## 2. Methods

### 2.1. Study Subjects

Ethical approval was acquired from Seremban hospital as well as Universiti Putra Malaysia with reference number [UPM.FPSK.PADS/T7-MJETIKAPer/F01-JSB-Mac]. All participants were asked to fill in informed consent questionnaires. In the current study, 320 Malaysian participants were interviewed. The blood pressure was measured on the right arm (three times) and averaged after resting (5 min intervals) by digital Sphygmomanometer on the basis of inclusion criteria [SBP above 140 mmHg and DBP above 90 mmHg (for patients)]. Participants' height and weight were taken to measure BMI utilizing the formula [weight (kg)/height (m^2^)]. All participants had biochemical analyzing test using plasma electrolytes.

### 2.2. Genomic DNA Extraction

In the study, the buccal and blood cells were collected from cases (hypertensive patients) and controls, respectively. The blood was kept in ethylene diamine tetra acetic acid (EDTA) tube and stored at 4°C for a maximum of three days before utilizing. The DNA was applied for amplification after extracting it from buccal and blood cell samples using Qiagen kit (Germany); then it was stored at −20°C for later usage. DNA was qualified right after all primers were optimized by PCR method. By utilizing the Nanodrop in two optical density (OD) wavelengths (260 nm and 280 nm), the extracted DNA concentration was examined.

### 2.3. Biochemical Analysis

In this section, peripheral venous blood samples were collected after keeping an overnight fast by control subjects. Moreover, lipid profiles [including TG, LDL, total cholesterol (TCH), and high-density protein (HDL)] were determined. We also measured FBS with standard laboratory techniques; however, the diabetic glucose levels were taken from the medical history. It was noticeable that we had referred to the hospital patients' documents to assess biochemical information for cases.

### 2.4. Determination of WNK4 Gene Ala589Ser Polymorphism

#### 2.4.1. Polymerase Chain Reaction (PCR)

Genomic DNA was amplified by multiplex-PCR reactions. The examined polymorphism of WNK4 (Ala589Ser) was identified using PCR based RFLP. PCRs were carried out with forward primer 5′-TGGAAACCCATTTTCCCCTGG-3′ and reverse primer 5′-AGGTGGTGAGGCCTAGAAAGT-3′ at the specific temperatures. Each reaction was composed of 6x master mix (which embodies DNA polymerase, MgCl_2_, dNTPs, and reaction buffers), 0.6 *μ*L relative primers (0.3 *μ*L forward and 0.3 *μ*L reverse), and 1 *μ*L of genomic DNA and ultimately distilled water was added to a final volume of 25 *μ*L. The DNA was amplified using initial denaturation of 94°C (5 minutes), followed by 40 cycles of 55°C annealing temperature for WNK4 gene polymorphism (90 seconds), 72°C extension (60 seconds), and kept at 72°C (10 minute) for final extension. Amplified PCR products were analyzed by gel electrophoresis methods with agarose.

#### 2.4.2. Restriction Fragment Length Polymorphism (RFLP)

The enzyme* AlwNI* was used for restriction of the PCR products. A total of 10 *μ*L PCR products were incubated at 37°C for at least 3 hours. The restriction enzyme and corresponding buffer (5 *μ*L) were added to the final restriction volume. After incubating, the achieved digested PCR product proceeded for electrophoresis on 2% agarose gel at 100 bp DNA molecular weight markers. The following electrophoresis was observed to determine the genotype of WNK4 gene Ala589Ser polymorphism. The size of the PCR product could be determined comparing with a 100 bp DNA ladder which was used as a DNA marker. Finally, the gel was washed with water for few minutes before viewing under Alpha Imager.

### 2.5. Statistical Analysis

The statistical analysis in present study was conducted by statistical package for the social science (SPSS version 22). Utilizing two-tailed Student's *t*-test and one-way ANOVA test, all variables among the groups and the group means were being contrasted (*p* < 0.05 was viewed statistically significant). The distribution of genotype with Hardy-Weinberg expectations was calculated using a Chi-squared test and allelic frequencies were analyzed by gene-counting method. In order to detect the effects of high risk alleles, odds ratios (OR) with 95% confidence intervals (CI) were checked as well.

## 3. Results

### 3.1. Clinical Characteristics

In the study, 320 volunteers, divided into hypertensive (163) and normotensive (157), participated for screening Ala589Ser polymorphism in WNK4. The clinical characteristics mean and standard deviation (SD) were clearly indicated in Table 1; however, the EHT mean age (59.07 ± 10.44) was noticeably higher in contrast to the controls (52.51 ± 9.41). Considering age, SBP, DBP, BMI, FBS, LDL, and TG, the subjects' clinical characteristics illustrated significant differences for most parameters between hypertensive and normotensive subjects (*p* < 0.05). Excluding HDL and TCH, other blood pressure related characteristics and the Ala589Ser polymorphism in WNK4 exon 8 were found to be related with elevated risk for the disorder (Table 1). Table 1 also shows significant differences for T2DM between hypertensive and normotensive subjects (*p* < 0.05).

### 3.2. Genotype and Allele Frequency

**Figure 1:**
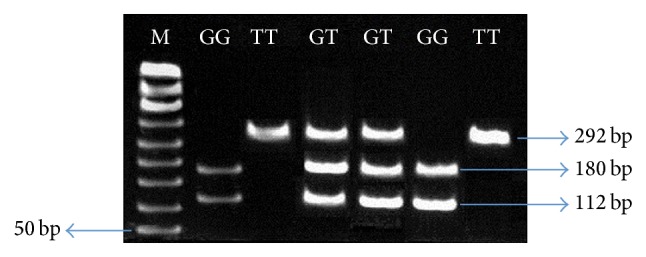
Detection of Ala589Ser polymorphism of WNK4 gene by PCR-RFLP utilizing 2.0% agarose gel electrophoresis. PCR products were digested by AlwNI. Wild type was cut (into 180 and 112 bp), while variant type remained uncut. Homozygous genotypes are indicated as GG genotypes, heterozygous genotypes are determined as GT genotypes, and TT genotypes show mutant genotypes. M represented a 50 bp DNA ladder plus (Bioline).

Multiple PCRs were used to determine the absence and presence genotype of Ala589Ser of WNK4 gene in samples. Before doing main RCR, clarity of the bands was checked by optimization of the method. Gene was set up separately with internal control to find out the sharp band. Each gel illustrated the optimization on one gene with four different samples. PCR products were run on 2% gel with four different samples; 50 bp ladder was used as a DNA marker. After PCR product digestion by* AlwNI* restriction enzyme, the gene encompassing Ala589Ser variant was detected through RFLP method using 2% agarose gel electrophoresis. The enzyme digested the variant type alleles in two fragments of A589 that was divided into 180 and 112 bp, whereas wild type (S589) remained uncut.  Figure 1 shows the Ala589Ser polymorphism amplification product of WNK4 gene.

Table 2 shows the WNK4 gene, Ala589Ser polymorphism, distribution between hypertensive and normotensive subjects. Homozygote (GG) genotype calculated the highest frequency where case and control were 95 (58.3%) and 109 (69.4%), respectively, while lower frequencies were observed in heterozygote (GT) genotypes in case 57 (35.0%) and control 48 (30.6%). Interestingly, mutant (TT) genotype determined the lowest frequency in cases which was 11 (6.7%) but not in the controls. Significant differences in genotypes were detected between cases and controls.

In Table 2, G allele was found to be higher in the control subjects which was 266 (84.7%) as compared to patients,while T allele was higher inpatients. Significant differences in genotypes and allele frequencies were observed between cases and controls with *p* value of 0.005 and 0.015, respectively (*p* < 0.05). Significant values were specified by one-way ANOVA with post hoc test for genotypes TT versus GG (0.001) and TT versus GT (0.004) between hypertensive and normotensive subjects; however, no significant value was obtained for TG versus GG. No significant difference was achieved from the gender and race's genotype for WNK4 gene Ala589Ser polymorphism within genotypes of cases and controls.

### 3.3. Genotype-Clinical Characteristics Correlations

To specify genotype-clinical characteristics correlations, clinical characteristics of patients between genotypes of Ala589Ser polymorphism were contrasted (Table 3). One-way ANOVA was applied to analyze the relation of clinical characteristic factors within genotype groups in patients. The WNK4 T allele established no relation with the genotype of the clinical characteristics of patients. It must be noted that no difference was identified in clinical disability in regard to WNK4 Ala589Ser gene polymorphism.

### 3.4. DNA Sequencing

In order to confirm the genotyping results, random samples were used and repeated with the same PCR conditions. To receive a final confirmation of the nucleotide sequence, purified PCR products were sent to Research Biolabs Malaysia. The sequencing results were aligned with the gene sequence from the NCBI-GenBank by the MEGA4 software [16].

## 4. Discussion

We targeted Ala589Ser polymorphism of WNK4 as a candidate gene in hypertension to specify potential association affecting high blood pressure determination. We specified a human-specific polymorphic Ala589Ser in WNK4 exon 8. In spite of the fact that the relevant research on Ala589Ser polymorphism variation of WNK4 gene was rather basic, close relation of WNK4 gene between cases and controls was recognized well recently [13]. Sun et al. indicated close relation between Ala589Ser polymorphism and both increased SBP and DBP. In addition to kidneys, WNK4 gene expression was observed in several other organs as well [13]. Kamide and colleagues identified 3 novel missense mutations of WNK4 gene in hypertensive patients among Japanese population [17]. The studies by Cao et al. (2010) on Kazakhs ethnic group in Xinjiang proposed that Ala589Ser polymorphism of WNK4 gene exhibited relation with high blood pressure and the T allele possibly produced risk factor for EHT [18]. In this study, genotype and allele frequencies of Ala589Ser showed significant association with EHT (*p* = 0.004, *p* = 0.003).

Although some researchers as Lu in china and Lv in Uyghur ethnicity did not observe significant association of Ala589Ser for genotype and allelic frequency [19, 20], research conducted by Erlich and colleagues on Ala589Ser of WNK4 demonstrated that there was no association in genotype or in allele frequencies with hypertension among African American population [21].

Guo et al. (2014) also pooled five studies in their meta-analysis; they managed to illustrate significant association between Ala589Ser of WNK4 and hypertension using both dominant genetic (OR = 51.85, 95% CI: 1.07–3.19, and *p* = 0.03) and allele contrast models (OR = 51.62, 95% CI: 1.11–2.38, and *p* = 0.01) [22].

WNK4 is expressed mainly in collecting ducts and distal convoluted tubules of the kidney and colon. Ala589Ser polymorphism of WNK4 is mapped in* Homo sapiens* chromosomes 17 [23]. The recent studies demonstrated that the equilibrium between potassium secretion (K^+^) and sodium chloride (Na^+^Cl^−^) reabsorption was regulated through WNK4 (both in vitro and in vivo) as a molecular switch by modulating the effect of the paracellular chloride (Cl_2_) pathways, the renal outer medullary potassium (ROMK) channel, the epithelia sodium channel (ENaC), and the Na^+^Cl^−^ cotransporter (NCC) [22, 24–26]. Expression of NCC increase in distal convoluted tubule (DCT), enhancement in paracellular Cl_2_ permeability of the distal tubule, and the decrease in ROMK channels surface expression might be induced by mutations of WNK4 [22, 25, 27, 28]. Hypertension development is also affected by NCC overactivity caused by Na^+^ retention in the DCT. Moreover, the results of this case control study confirmed the association of WNK4 gene variation with the susceptibility of hypertension.

The present study perhaps is the first effort to specify the relation between WNK4 gene Ala589Ser polymorphism and EHT in Malaysia. Significant association of Ala589Ser polymorphism, WNK4 gene, and hypertension has been specified in Malaysian genotype. Close association between TT genotypes/T allele of WNK4 gene and EHT has been demonstrated in contrast to controls in Malaysia (*p* < 0.05). Statistically, substantial differences have been observed between EHT and controls (*p* = 0.000) which matched Chinese (*p* < 0.035) and African American population (*p* < 0.05) [10]. The genotype distribution with Hardy-Weinberg expectations was calculated using a Chi-squared test and allelic frequencies were analyzed by gene-counting method. Odds ratios with 95% confidence intervals were checked in order to determine the influence of high risk alleles.

Considering the fact that 88 individuals out of 163 hypertension cases were diagnosed with T2DM based on their hospital records, a strong relation was established between hypertension with T2DM and Ala589Ser (*p* < 0.05) in the study. To reveal a relation between T2DM and Ala589Ser polymorphism, I made a careful analysis which was the novelty of this study. I divided the case group into EHT and EHT+T2DM. Significant differences in genotypes and allele frequencies were observed between EHT patients and control group with *p* value of <0.001 and <0.017, respectively (*p* < 0.05). In hypertensive subjects with T2DM, the significant record was shown for their genotypes and allele frequencies which were 0.004 and 0.009, respectively, in comparison with controls (*p* < 0.05) (Table 4).

Significant differences in age, SBP, DBP, BMI, FBS, LDL, and TG were found between hypertensive and normotensive subjects (*p* < 0.05), although HDL and TCH levels showed no significant differences in hypertensive subjects and controls (*p* > 0.05). Significant differences of clinical characteristics (BMI, HDL, LDL, TG, TCH, SBP, and DBP) were identified between healthy individuals and EHT as reported previously [13]. Interactive influences were also observed by Cao et al. between WNK4 gene Ala589Ser polymorphism, BMI, and gender [18].

In summary, we analyzed Ala589Ser polymorphism of WNK4 gene. The Ala589Ser polymorphism displayed significant association between genotype/allele frequency and hypertension; this polymorphism has a potential effect on the clinical characteristics profile of the subjects. According to Sun et al., different hormones might have effect on the WNK4 gene expression in the kidneys. Increasing susceptibility in essential hypertension may be significantly influenced by the Ala589Ser polymorphism as a missense mutation [13].

The findings of the present research indicate that TT genotypes/T allele of WNK4 gene produce a close relationship with EHT and T2DM. The current research must be interpreted within its context restrictions. The proof of the relationship between T/G variant of WNK4 gene and EHT at the gene level has been provided only; nevertheless, the function and mechanism of T variant have not been addressed. Second, in contrast to cases, the control subjects are relatively young but not age matched; however, more relative studies with larger population concerning other candidate gene polymorphisms or T/G polymorphism of WNK4 gene in relation to cardiovascular disorder are suggested.

## Figures and Tables

**Table 1:** Baseline characteristics of the subjects ±.

Parameter	Hypertensive subjects	Normotensive subjects	*p* value
Gender (male/female)	(90/74)	(73/83)	NS
Age (year)	59.07 ± 10.44	52.51 ± 9.41	<0.0001^*∗*^
SBP (mmHg)	149.11 ± 21.00	122.21 ± 11.23	<0.0001^*∗*^
DBP (mmHg)	86.02 ± 9.51	76.18 ± 8.76	<0.0001^*∗*^
BMI chol (mmol/L)	27.09 ± 5.08	24.80 ± 3.68	<0.0001^*∗*^
FBS chol (mmol/L)	6.37 ± 1.15	4.88 ± 0.58	<0.0001^*∗*^
T-chol (mmol/L)	4.71 ± 0.98	4.88 ± 1.31	NS
LDL-chol (mmol/L)	2.69 ± 0.97	3.21 ± 1.10	<0.0001^*∗*^
HDL-chol (mmol/L)	1.26 ± 0.32	1.28 ± 0.52	NS
TG (mmol/L)	1.55 ± 0.52	1.22 ± 0.55	0.0001^*∗*^
Diabetes (yes/no)	(88/75)	(19/138)	0.000
Family history (yes/no)	(73/89)	(2/155)	0.000
Smoking habits (yes/no)	(30/133)	(13/144)	0.008
Alcohol drinking (yes/no)	(23/140)	(2/155)	0.000

Data are mean ± SD unless otherwise specified. ^*∗*^The *p* value of genotypes and alleles was calculated using *t*-test. NS: not significant.

**Table 2:** Genotype and allelic frequency distribution between hypertensive and normotensive subjects.

	Hypertensive subjects	Normotensive subjects	*p* value
Genotype			
GG	95 (58.3)	109 (69.4)	0.005^*∗*^
TG	57 (35.0)	48 (30.6)	
TT	11 (6.7)	0 (0.0)	

Allele frequency			
G	247 (75.8)	266 (84.7)	0.015^*∗*^
T	79 (24.2)	48 (15.3)	

Post hoc test	*p* value	(95% confidence interval)	

TT versus TG	0.004	0.15–0.76	
TT versus GG	0.001	0.23–0.83	
TG versus GG	0.193	−0.19–0.4	

^*∗*^Significant value (*p* < 0.05) was achieved through Chi-square test as compared with controls. Data were reported in number with percent in parentheses. EHT in comparison with control subjects.

**Table 3:** Genotype-clinical characteristics correlations in patients.

Variable	GG	GT	TT	*p* value
Gender (male/female)	52 (54.7%)/43 (45.3%)	31 (54.4%)/26 (45.6%)	7 (63.6%)/4 (36.4%)	NS
Age (year)	55.63 ± 10.21	55.83 ± 10.73	60.27 ± 12.49	NS
SBP (mm/Hg)	134.97 ± 21.50	135.99 ± 22.02	152.73 ± 11.69	NS
DBP (mm/Hg)	80.25 ± 9.81	82.87 ± 11.22	82.91 ± 10.96	0.041
BMI (cm/kg)	25.83 ± 4.93	26.24 ± 3.92	25.87 ± 4.10	NS
FBS (m/mole)	5.51 ± 1.09	5.87 ± 1.31	5.87 ± 1.01	NS
TCH (m/mole)	4.87 ± 1.26	4.64 ± 0.96	4.75 ± 0.72	NS
LDL (m/mole)	3.01 ± 1.13	2.82 ± 0.95	2.91 ± 0.94	NS
HDL (m/mole)	1.30 ± 0.46	1.21 ± 0.36	1.34 ± 0.35	NS
TG (m/mole)	1.39 ± 0.56	1.38 ± 0.56	1.34 ± 0.51	NS
Diabetes (no/yes)	46 (48.4%)/49 (51.6%)	22 (38.6%)/35 (61.4%)	7 (77%)/4 (44%)	NS
Family history (no/yes)	42 (44.7%)/52 (55.3%)	23 (40.4%)/34 (50.6%)	8 (72.7%)/3 (27.3%)	NS
Smoking habit (no/yes)	77 (81.1%)/18 (18.9%)	48 (84.2%)/9 (15.8%)	8 (72.7%)/3 (27.3%)	0.045
Alcohol consumption (no/yes)	86 (90.5%)/9 (9.5%)	44 (77.2%)/13 (22.8%)	10 (90.9%)/1 (9.1%)	NS

Data are presented as mean ± SD; *p* value > 0.05.

**Table 4:** Genotypes and allele frequencies distribution of gene polymorphisms between two patient groups and control subjects.

	EHT *n* (%)	EHT+T2DM *n* (%)	Control *n* (%)
Genotype and allele frequency			
GG	46 (61.3)	49 (55.7)	109 (69.4)
GT	22 (29.3)	35 (39.8)	48 (30.6)
TT	7 (9.3)	4 (4.5)	0 (0)
G	114 (76)	133 (75.6)	266 (84.7)
T	36 (24)	43 (24.4)	48 (15.3)
*p* value	0.001^*∗*^/0.017^*∗*^	0.004^*∗*^/0.009^*∗*^	
Odds ratio (95% CI)	571 (252–928)	0.558 (0.352–885)	

Post hoc test			

TT versus GT	0.000	0.018	
TT versus GG	0.000	0.004	
GT versus GG	0.790	0.083	

^*∗*^ *p* value < 0.05.
